# Remarks on Tin and Gold or “Tg”

**Published:** 1888-04

**Authors:** C. M. Wright


### Remarks on Tin and Gold, or "Tg."

BY C. M. WRIGHT.

Mr. President:—I have here in my hand a lecture published in Germany, and in the German language, from Dr. W. D. Miller, Professor in the Dental Institute of the University of Berlin,—which Dr. Miller sent to me after he had received the programme of subjects of this society. It is on the "Combination of Tin and Gold as a Filling Material for the Teeth."

It is a very interesting discourse on the subject, from one who has had unusual opportunities for observing the practical results of the combination of metals in the teeth of, I dare say, many thousands of patients. The lamented Dr. Abbot, of Berlin, had employed the combination for twenty-five years introducing it with many encomiums to his friends, and Drs. Jenkins of Dresden, and Sachs of Breslau, have employed it for a score of years and are still enthusiastic, if such a word can be used in expressing the convictions of such cautious scientific observers and careful practitioners as these, in its praises. It has, I may say, been quite extensively employed as an invaluable aid to operative dentistry by many eminent American and German dentists on the continent of Europe for many years. The interest taken in this subject of combination fillings is a comparatively new one in the United States. While here and there a man has employed tin foil in the bottom of a cavity and gold foil as the covering of the tin, in this country, I think we must admit that it has not become a recognized practice to systematically combine tin and gold for a special purpose and as a special material for filling special kinds of carious teeth. A few dentists here and there in this country have introduced the "Tg." upon their operating tables, but it is by no means universal. Now these gentlemen in Europe and these gentlemen in America who are known to have adopted this system in practice, have not done so for economical reasons. It is not a question of saving in the amount of gold. What then are the reasons offered for its use as a filling material? If we turn to Professor Miller's brochure we find two reasons given. One is the ease afforded to both operator and patient in the introduction of a tight or perfect filling, and the other is the number of valuable qualities which the combination affords as a material. These qualities are 1st, durability, equal to gold alone. 2d, non-conductible; it is superior to gold alone and equal to tin foil, 3d, the peculiarity of this special combination of metals, from change which it undergoes in the tooth in the mouth after its mechanical introduction there—these changes affecting a secondary combination of the metals and producing a filling which in a short time becomes harder in the cavity than when first introduced.

To those of you who have never filled a tooth with "Tg." and then have never had the opportunity of cutting out this same filling a year afterwards, this latter quality of the secondary changes has no meaning, but to those who have and who know that the soft tin and soft gold introduced as sheets to-day, may become as crisp and hard to the excavator as a good amalgam, in the course of time, the *quality* of which we speak is a distinct one and belongs to no combination of metals with which I am acquainted, excepting those in which mercury is a constituent.

Dr. Miller in his brochure details clearly his own, Dr. Jenkins and Dr. Sachs' methods of employing the material in the various cavities, and also methods of covering the "Tg." with gold, where this is desirable, (desirable not on account of hardness and durability but on account of appearance only.) The book is fully and uniquely illustrated, and I shall take pleasure in passing it around for your inspection of the figures which form clear diagrammatic lectures in themselves of the entire process which can be readily grasped by the experienced operator at a glance. (The figures were drawn by Dr. Adolph Witzel.)

In regard to this change which takes place in old fillings of the combination, Dr. Miller offers the following experiments and results. He repeats first: The mass of filling is found to have become hard and stone-like in character, and often in cutting into it, is liable to be mistaken for an amalgam filling by the inexperienced dentist, or rather by the dentist who meets this filling for the first time in a tooth.

We have been in the habit of saying that the change in color of the "Tg." filling is on account of the oxidation of the tin, but when we remember that the oxide of tin is white and not black we may well suspect that we have not made a correct statement of the real cause of the discoloration.

If you will lay a sheet of gold foil between two sheets of tin foil and place them in a one per cent. solution of lactic acid, and keep this at the temperature of the blood of the body for a few hours, at the end of this time you will hardly be able to distinguish the gold from the tin. The gold will have lost its yellow color and become gray like the tin. A few hours later the gold will have become brownish, and the whole mass will have become bronze or nearly black in color. In the solution a white flakey deposit will also be found which consists of the oxide of tin. On the gold the deposit, or layer of gray black, consists of the metalic tin. A great deal of tin is found also in the solution itself, but no gold in any appreciable quantity. This follows very quickly in a solution of lactic acid—which is a condition easily believed to be present in the mouth. This change in the metals is not found so readily when the experiment is made with acetic acid, and not found at all in a solution of butric acid. It is also found in solutions of hydrychloric and sulphuric acids, but not at all in a solution of nitric acid.

If these experiments are made under pressure, that is, when the sheets of tin and gold are pressed together, the additional phenomenon of an adhesion of the two metals seems to occur, and the gold and tin cannot be separated. If one layer of gold is placed upon a layer of tin, only the under surface of the gold discolors while the upper remains bright. If a glass plate is laid over the gold, however, even the upper surface of the gold will discolor but not so rapidly. Doctor Miller gives the following explanation of the phenomena:

In the weak lactic acid solution an electric current is established between the gold and the tin. The gold is electro negative to the tin. Hydrogen accumulates on the surface of the gold, and oxygen on the surface of the tin. The oxygen unites with the tin and forms oxide of tin. The electro-positive oxide of tin meets the electro-negative gold, where it is deprived of its oxygen by the hydrogen found there and is thrown down as metallic tin on the surface of the gold. He accounts in this way for the facts that when the upper surface of the gold is uncovered it remains bright, because the hydrogen disappears from this surface as soon as formed and no reduction of the oxide of tin occurs, and that under an indifferent cover, like glass, hydrogen is, in a degree, prevented from escape, and so the surface of the gold becomes slowly colored by the same process.

The same thing occurs in the mouth, and yet while the gold seems entirely to have disappeared and a trace of it cannot, in some cases, be found by the microscope in old filling, still by a prolonged treatment of the filling with nitric acid it is found not to have been changed and gold can be recovered.

Another point of practical importance to the dentist is that when the tin and gold have become moistened before the experiments with the acids the entire action seems to be hastened, and this is practically shown by fillings in the mouth. Moisture is not detrimental to this combination as it is to all other materials which we employ for the filling of teeth, and in nervous patients where great irritability or sensibility exists in the mucous membrane of the gums, or tongue, or palate, and where even the contact of bibulous paper, or the finger, or the napkin, or rubber dam causes distress, a tin and gold filling can be successfully made even in the presence of considerable moisture and yet possibly be a better filling than one introduced in a very dry state, as the electrical changes are hastened and hardening occurs sooner in the former than in the latter. Careful experiments have been made by Witzel and others in regard to this point by dipping the first pellet of the "Tg." in different solutions, and afterwards covering them with dry pellets and pressing them in, so that the moisture would penetrate the filling, and observing the rapidity of the changes in hardening, etc.

One word more. In regard to the late hypothesis offered by an American dentist in a late journal on the possibility of an electric current being established between two metals in one cavity which is capable of killing by lightning the poor little bacteria which may happen to be hiding in the neighborhood of the combination filling, Dr. Miller has not expressed an opinion in his book, but if the society will keep my words to themselves, I will repeat that in a private postal card Dr. Miller has expressed his belief that the lightning in this case need not scare the bacteria from the neighborhood, as it can have no greater effect upon them than a " bread pill would have upon a rhinoceros."

DISCUSSION.

*Dr. Wright:* Personally have had some fifteen years experience with "Tg.", and in its use prefer the roll of tin and gold to be twisted or rolled into a rope with the gold and tin showing like the stripes on a barber pole, in equal parts; this separates it more easily from pellets of gold alone, or cylinders of gold alone. Have used it in cylinders made by rolling a strip of gold and tin with the tin inside sometimes and sometimes outside. Use this always without the dam. Have also used "Tg." at the cervical borders of large proximal compound cavities with more satisfaction than any other material at these vulnerable points. Employ it in children's teeth to some extent when red gutta percha does not seem to meet the demands of the case.

Also find it a most valuable material for 6th year molars in young patients.

The only objections that can be brought against the combination is its color, that is, the color which it assumes after a while. It does not discolor the softest tooth, however, at all, (and its not being strictly a plastic filling). From careful observations for a series of years I must add my testimony in favor of the Tin-gold as an easy way of saving teeth by filling, and as having importaut qualities not posessed by gold alone or tin alone.

It is more easily inserted than non-cohesive gold even, and with less force and pressure becomes harder and more durable. That is if we should take the same quantity of soft gold and apply the same amount of force we should not make as perfect a filling as we would with this "Tg." The gold alone requires more pressure, for it must be made hard enough for use by firm packing, while a comparatively soft "Tg." filling will become hard enough to resist mastication by its own qualities.

*Dr. Cravens* had used the combination of tin and gold as an experiment in 1876 and 1877. He had replaced the fillings, for the material seemed to wear away, was black, and in some cases there was a tendency to form spaces by contraction at different points. The most practical method that he had found for using the two materials, was to use the tin in mass, and the gold in mass; filling the cavity partly full with tin and building the gold upon this. He thought the preservation by tin was due to its soft working qualities and adaptation to the inequalities of the cavity. He used it even on the palatine portion of incisors.

*Dr. Sage:* I have noticed that some writers say that number two tin should be used with number four gold to get just the proper combination; but writers differ on this point and the statements become confusing. We must have no rebounding of the instrument, but have the material in such condition that it will stay where it is put. It seems to me that we are also in error in building up with cohesive gold, for the attachment is not reliable unless undercuts are made.

*Dr. Conrad* said that a short time since Dr. Eames had a patient of Dr. Herbst's. There were about fifty of these combination fillings in the mouth. In ten or twelve the tin had disappeared entirely, in two or three partially, and the other fillings were in such a condition that they all had to come out. The failure was attributed to the kind of tin used in the operations.

*Dr. Betty* requested that all old gold-and-tin fillings be sent to him for analysis and investigation.

*Dr. Watt* said that in some cases the action brought about by the combination caused uneasy sensations or pain in the tooth and sometimes so severe as to cause sleeplessness. He stated that it was not thoroughly understood just what the trouble was, but he was of the opinion that it was *thermal electricity*. He reported a case where a tooth was replanted and regained complete nerve union.

*Dr. McKellops* had seen much of the combination filling, but there was nothing that gave him more satisfaction than gold. He thought non-cohesive gold was just as adaptable as tin or tin and gold. This could be proven by experiment. Take tin and adapt it to the wall of a cavity, and then adapt pure gold to the same wall, and you will perceive that the hair lines on both are precisely alike. He often used very large cylinders of No. 4 noncohesive gold with profit.

*Dr. Taft:* We ususually have less gold en masse under the plugger than of the tin; and while it can be as perfectly adapted as tin, it does not work quite as easily and requires more time.

